# Supplementary material for: Pre-trained Convolutional Neural Networks Identify Parkinson’s Disease from Spectrogram Images of Voice Samples
Source: Res Sq. 2024 Dec 18:rs.3.rs-5348708. Preprint. [Version 1] doi: 10.21203/rs.3.rs-5348708/v1 (PMC11702857; doi:10.21203/rs.3.rs-5348708/v1)
Supplement: Supplement 1 [file NIHPPRS5348708v1-supplement-1.pdf]

Supplementary Files

This is a list of supplementary files associated with this preprint. Click to download.

- SupplementaryFile1.pdf
- TableS1.csv
